# Development and psychometric validation of the Colorectal Cancer Literacy Scale—Uruguay Version

**DOI:** 10.3389/fpubh.2023.1179792

**Published:** 2023-08-08

**Authors:** Lydia P. Buki, Micaela Reich, Jue Wang, Selva Sanabia, Dolores Larrosa, Bibiana Sologaistoa, Mercedes Blanco

**Affiliations:** ^1^Department of Educational and Psychological Studies, University of Miami, Coral Gables, FL, United States; ^2^Departamento de Bienestar y Salud, Universidad Católica del Uruguay, Montevideo, Uruguay; ^3^Area de Educación Poblacional, Comisión Honoraria de Lucha Contra el Cáncer, Montevideo, Uruguay

**Keywords:** health literacy, psycho-oncology, Latin America, cancer control, scale development

## Abstract

Colorectal cancer is a leading cause of cancer death in Uruguay, yet less than half of the eligible population is up to date with screenings. Research is hampered because no measures exist to assess psychosocial factors that influence screening decisions in this population. To address this gap, we report on the development and psychometric validation of the Colorectal Cancer Literacy Scale—Uruguay Version, a scale based on the health literacy model. We developed an item pool based on the extant literature, obtained feedback from experts, and conducted focus groups with community participants and health care providers. After revision, we conducted a psychometric validation with a national community sample of 405 participants. Through an exploratory factor analysis, we identified four factors that were collapsed into two for theoretical and pragmatic reasons, representing (a) disposition toward cancer prevention and (b) attitudes, beliefs, and emotions about cancer. A third factor, knowledge about colorectal cancer, was examined separately given its distinct focus. Subsequently, we conducted a confirmatory factor analysis with the remaining sample participants using Rasch measurement theory for validation purposes and to further assess the scales’ psychometric properties. The resulting 44-item scale presented a good model fit with adequate EAP reliabilities and good initial discriminant validity. Further criterion-related validity analyses should be performed when additional measures are available. The Colorectal Cancer Literacy Scale—Uruguay Version is a theoretically based measure that can bring to light barriers and facilitative factors in an underscreened population at risk. Implications for theory, research, and practice are discussed.

## Introduction

Colorectal cancer (CRC) is a leading cause of cancer burden among men and women, with approximately 2 million people diagnosed per year worldwide (1). This is a critical public health issue, given that CRC can be prevented through routine screening (2). Importantly, as individuals adopt lifestyle behaviors that enhance cancer risk (e.g., low-fiber diet, sedentarism), incidence and mortality rates are increasing in populations under the age of 50 (3, 4).

Uruguay, with a growing economy and with one of the most stable democracies in the Americas (5), shares globalization trends related to diet and lifestyle, yielding negative public health outcomes. Specifically, among cancers, CRC is a significant concern in Uruguay—its incidence and mortality rates are consistently ranked among the highest in the Americas (6). In Uruguay, across all cancers, CRC incidence and mortality rates rank second-highest for women (25.53 and 11.89 per 100,000, respectively) and third-highest for men (37.12 and 18.48 per 100,000, respectively (7). In addition, mirroring global trends, rates are on the rise for individuals under 50 years of age (8). These statistics are particularly concerning because through routine fecal occult blood tests (FOBT), it is possible to detect precancerous lesions and malignant tumors at early stages, when survival rates are highest (9). However, due in part to low screening rates (which, in turn, lead to late detection), morbidity and mortality remains high in the population.

Only 42% of Uruguayans between the ages of 50 and 64 have obtained the FOBT, despite the fact that the Ministry of Public Health recommends biennial FOBT screening for individuals 50–74 years of age (10). The fact that over half of eligible patients is not screening is a public health issue in need of attention. Moreover, given that the Uruguayan health care system has a public safety net (11, 12), the FOBT can be obtained, generally, for low cost. Thus, it is critically important to understand possible additional deterrents to screening. Psychosocial factors are likely to play a prominent role in individuals’ decisions not to engage in preventive behaviors, yet there are no measures available to assess these risk factors. To fill this research gap, the goals of the current study were to (a) develop a scale that would measure psychosocial influences on CRC screening behaviors in Uruguay, and (b) conduct a national study to assess the scale’s psychometric properties. With information about psychosocial determinants of CRC screening, interventions may be designed to effectively promote early detection, optimize public health resources, and lower the large financial burden of comorbid conditions and premature deaths due to CRC.

### Conceptual framework

Research suggests that psychosocial factors affecting CRC screening may be nonmodifiable, such as SES and formal educational attainment (13), or modifiable, which are of special interest to health service providers. Modifiable factors include dietary and lifestyle patterns (14) as well as knowledge, beliefs, attitudes, and emotions about cancer and screening (15). The latter are amenable to intervention and are the focus of the current study.

A fitting conceptual framework to examine modifiable factors that influence screening uptake is health literacy. Defined as “the degree to which individuals have the capacity to obtain, process, and understand basic health information and services needed to make appropriate health decisions” (16), individual health literacy is theorized to promote taking an active role in one’s health. In fact, there is documented evidence of an association between health literacy and CRC screening [e.g., (17–19)]. In an investigation conducted as part of the English Longitudinal Study of Aging, individuals with higher levels of health literacy had 20% greater odds of participating in a national CRC screening program than those with lower health literacy levels (20). Similarly, other national and international studies have reported links between inadequate health literacy and greater barriers to, and lower rates of, CRC screening [e.g., (18, 21–23)]. Several mechanisms may underlie health literacy’s influence on health outcomes. For example, individuals with low health literacy have reported (a) greater barriers related to seeking and reading health-related information, and (b) lower CRC screening self-efficacy (24). Given these empirical associations, we set out to extend knowledge about psychosocial factors that influence CRC uptake in Uruguay based on a health literacy framework.

Health literacy is a complex construct that encompasses several components, namely print and oral literacy, numeracy, and cultural and conceptual knowledge. The latter represents the filter through which individuals obtain, process, and understand health information and options for diagnosis and treatment (25). Factors that compose cultural and conceptual knowledge include knowledge, beliefs, attitudes, and emotions (26). Data on these factors are needed as a foundation for the design of effective interventions and to influence policy [e.g., (27, 28)]. Based on the health literacy model, cultural and conceptual factors are posited to “reside” in the individual, yet they develop and interact within various larger contexts including culture and society, the health care system, and the educational system—which, in turn, are considered points of intervention (25, 27).

A growing evidence base suggests that aspects of cultural and conceptual knowledge, specifically, are related to cancer screening behaviors (23, 26, 29, 30). Despite evidence of this link, as well as Uruguayans’ higher risk for CRC, we could not identify any studies on CRC health literacy conducted in Uruguay. A handful of studies published on CRC have focused on the influence of diet or genetic profiles on incidence rates [e.g., (31, 32)]. The limited literature base focused on psychosocial factors precludes empirical advances in this critical area.

Therefore, given the importance of assessing cultural and conceptual knowledge and its influence on CRC screening in Uruguay, a psychometrically valid scale is needed. In this effort, we developed the Colorectal Cancer Literacy Scale–Uruguay Version (CCLS–U), a new tool to assess cultural and conceptual knowledge related to CRC screening behaviors in Uruguay. In this article, we report on the process of scale development and present psychometric data from a national validation study. We begin with a review of the extant literature on factors that influence CRC screening in United States and international samples.

### Psychosocial factors that influence CRC screening

Studies focused on Latinx, non-Latinx Whites and other Black, Indigenous, and People of Color in the United States have identified numerous factors that negatively influence screening rates: (a) low health literacy [e.g., (33)]; (b) limited knowledge about CRC and the purpose of cancer screening tests [e.g., (33, 34)]; (c) beliefs about the health care system, including lack of confidence in the system and mistrust of individual providers [e.g., (33)]; (d) negative attitudes, such as pessimistic attitudes about CRC survival (34, 35); (e) negative beliefs about CRC screening [e.g., (15, 33-35)]; (f) lack of recommendation from the primary care physician (34, 35); and (g) system-level barriers such as cost, medical insurance, and transportation (15, 34). In international studies, low knowledge emerged as a key barrier to screening in Singapore (36, 37), and positive attitudes about the FOBT was identified as a facilitative factor in Spain (38).

Within Latin America, in Argentina, an adjoining country that shares sociocultural characteristics with Uruguay, 87% of patients with health insurance indicated that they would not obtain a CRC screening unless their doctor recommended it (39). Consistent with previous studies, participants with more favorable attitudes toward doctors reported higher screening rates. Also in Argentina, findings from a national sample showed a widespread lack of knowledge about CRC, its symptoms, methods of early detection, and treatment (40). Next, we describe the process of developing the CCLS-U.

## Materials and methods

### Item development: measuring cultural and conceptual knowledge related to CRC

**Figure 1:**
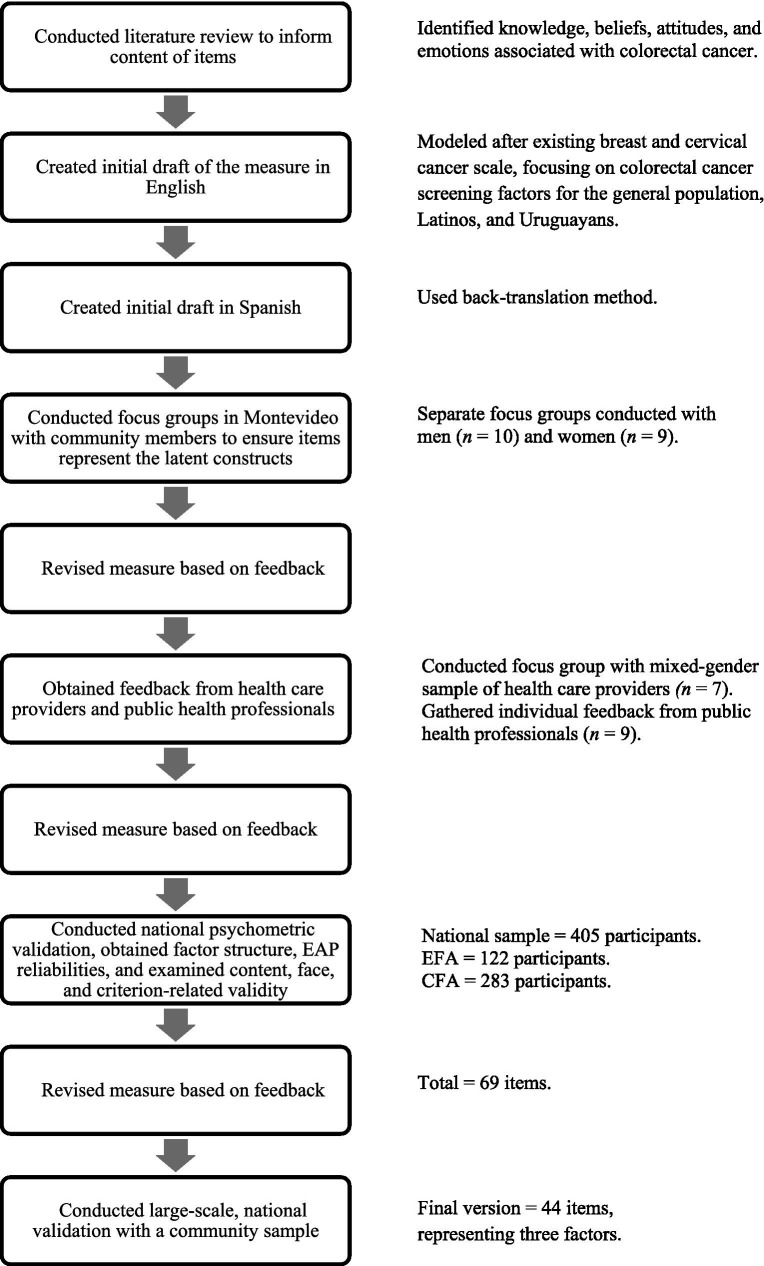
Overview of the scale development process.

For the present study, we conducted a literature review to inform the content of items related to knowledge, beliefs, attitudes, and emotions related to CRC. The CCLS–U was modeled after an existing scale designed to measure cultural and conceptual knowledge with respect to breast and cervical cancer in Uruguayan women (41). An initial draft was developed in English, subsequently translated to Spanish using the back-translation method. To create an initial pool of items, we sought to tap each critical aspect relevant to CRC, avoiding item constriction (42). Thus, we first identified potential factors influencing CRC screening behavior for the general population, followed by influences among Latinos in particular. We then adapted or developed items to further explore these potential determinants of screening behavior among the Uruguayan population. We also drew from the extensive knowledge of researchers and staff at the Comisión Honoraria de Lucha Contra el Cáncer, a public health organization that had conducted a national CRC health promotion campaign. Consistent with previous scale development studies in the area of cancer [e.g., (43)] and experts’ recommendations for scale development (44), we followed a mixed-methods approach and subsequently conducted focus groups to ensure drafted items covered a broad and representative item pool that would tap the full range of the latent construct. Figure 1 provides an overview of the scale development process.

### Item validation and cognitive debriefing: focus groups

Once the draft of the CCLS–U was developed, we conducted separate focus groups with a community sample of men (*n* = 10) and women (*n* = 9), and a mixed-gender sample of health care providers (*n* = 7), all of whom provided feedback on the scale. An exclusion criterion was having a personal history of CRC, as cancer survivors are likely to have different perceptions and needs than those without a diagnosis. The group interviews, which were held in Montevideo, Uruguay, in a private conference room with refreshments, were conducted in Spanish and lasted 90–120 min. We did not provide compensation for participation in the study. Informed consent was not required for this phase of the study, as participants were only asked to provide feedback on scale items.

#### Community sample

Participants were identified through community gatekeepers known to the staff of the Comisión Honoraria de Lucha Contra el Cáncer. Our goal was to recruit participants who represented a range of ages and formal education levels. On average, participants were 60 years old (*SD* = 7.85; *range*: 45–77 years) and had 15 years of formal education (*SD* = 2.80; *range*: 10–21).

Initially, we asked participants to read the scale draft and make written comments or highlight areas that were confusing or irrelevant to their experience. We then used an interview guide to facilitate a discussion about all items, inviting the participants to suggest revisions or additions based on their previous observations. Based on results from these focus groups, minor edits were made to the survey. For the demographic questions, we (a) added choices to certain items to reflect the national context with respect to health insurance and income options (e.g., we added two options related to health insurance providers), and (b) ensured that information sources mentioned by the participants were reflected among possible responses. In addition, we made one edit to an item measuring cultural and conceptual knowledge to separate one double-barreled question into two items.

#### Health professionals sample

We also obtained feedback from seven health care providers including three oncologists, two gastroenterologists, one endoscopy assistant, and an oncology nurse with expertise in CRC. We followed a similar procedure for this group, first inviting them to comment on the clarity and relevance of the items. We then asked them to propose additions related to any aspects of knowledge, beliefs, attitudes, and emotions based on observations from their medical practice. We combined their feedback with that obtained from nine public health professionals with expertise in the development of national cancer prevention campaigns in Uruguay, and made the following additional refinements to the demographic questions and scale items: (a) we rephrased three words to conform to linguistic usage in Uruguay (e.g., we changed “*colonoscopía*” to “*fibrocolonoscopía*”), (b) we revised response options for several items to conform to the sociocultural context in Uruguay (e.g., we added “Eating grilled red meat with charred fat” as a risk factor for CRC), (c) we added three items to reflect behaviors and beliefs related to cancer and CRC screening (e.g., “How important is it to get a colonoscopy if the doctor recommended it?”), (d) we changed some words to make the meaning more precise (e.g., we changed the word “mass” to “polyp”), and (e) we disaggregated a double-barreled question into two items. The final version of the CCLS–U comprised 69 items. We subsequently conducted a large-scale, national validation of the measure with a community sample.

### Participants

A total of 405 participants were recruited in community settings in five regions of the country (*departamentos*) akin to five US states: Colonia, Durazno, Lavalleja, Montevideo, and Rivera. We selected these *departamentos* due to their prominence in Uruguay, the fact that they represent various geographic regions of the country (i.e., Central, East, North, and South), as well as urban and rural settings. Inclusion criteria were: (a) born in Uruguay or having immigrated to Uruguay before the age of 11, (b) 50–74 years of age, and (c) no previous CRC diagnosis. The rationale for the inclusion criteria is as follows: we wanted to recruit participants who relied on the Uruguayan health care system and had a long history of exposure to cultural health beliefs in Uruguay. The age range mirrored the ages at which participants needed to screen per national guidelines, and individuals who had been diagnosed with cancer were not included due to their unique communication and knowledge needs.

Of the 405 participants, 80 were recruited in Colonia (41 women, 39 men), 81 in Durazno (42 women, 39 men), 80 in Lavalleja (40 women, 40 men), 80 in Montevideo (40 women, 40 men), and 84 in Rivera (43 women, 41 men). Participants were, on average, 60 years old (*SD* = 7; range: 50–74 years). Men and women exhibited diverse demographic characteristics. A little more than a third (37%) were no longer in the workforce. Formal education levels ranged from 0 to 29 years (*M* = 11.16; *SD* = 4.46; *Mdn* = 11.00). Annual household incomes ranged from 72,000 to 11,640,000 pesos (approximately $2,087–$337,391 USD; *Mdn* = $480,000 pesos). After taking out four outliers on the upper end of the distribution, participants’ incomes ranged from 72,000 to 1,800,000 pesos (approximately $2,087–$52,174 USD; *M* = 559,904.04 pesos; *SD* = 346,354.56 pesos). Most participants were married (53%), cohabiting (11%), or divorced (17%). About two-thirds of participants were members of a *mutualista* (a nonprofit health insurance network with a broad range of benefits, similar to an HMO in the United States) and a third were members of *ASSE* (a network of medical services provided by the government’s public health system). Approximately two-thirds of participants (68%) had ever obtained an FOBT. Because the survey was administered verbally, there was only one missing data point for one participant.

### Measures

#### Background questionnaire

A background questionnaire included 10 demographic questions to gather information such as age, gender, level of formal education, marital status, type of health insurance, household income, and occupational status. In addition, it included 13 questions related, specifically, to CRC. For example, items asked whether the participant’s insurance covers the FOBT, whether they have a family history of CRC, whether they have ever attended a workshop on the importance of cancer screening, and their awareness about the colonoscopy exam.

#### CCLM–U

The measure, which has 69 items, measures knowledge (33 items; e.g., “A symptom of colorectal cancer is blood in the stool”), and attitudes, beliefs, and emotions (36 items; e.g., “I dislike talking about cancer,” “There are things I can do to avoid getting colorectal cancer,” “If I found out I had colorectal cancer, I would feel sad”). Knowledge items had three possible answers: “Yes,” “No,” or “I do not know”; beliefs were measured on a scale from (1) *strongly disagree* to (5) *strongly agree*, and most attitudes and emotions were measured on a scale from (1) *very little* to (5) *very much*. Items 23 and 28, which measure beliefs, were reverse coded such that higher scores indicate beliefs facilitative of screening. Similarly, all knowledge items were coded such that a higher score indicates an accurate answer.

### Procedure

Community gatekeepers associated with the Comisión Honoraria de Lucha Contra el Cáncer recruited participants in community settings at each *departamento*. They set out to recruit 40 women and 40 men in each location. Gatekeepers used their knowledge of the community and local organizations to (a) identify prospective participants, (b) attend community events and make announcements about the study, and (c) identify additional prospective participants through snowball sampling. After ascertaining inclusion and exclusion criteria, and after obtaining informed consent, the gatekeepers verbally administered the scale, entering responses electronically on a tablet. To ensure anonymity, we did not collect participants’ names, addresses, or other identifiable information. The tablet was password-protected, and used to gather data which subsequently was encrypted and available only to study staff. On average, it took 20 min to complete the measure.

## Results

We conducted an exploratory factor analysis (EFA) followed by a confirmatory factor analysis (CFA). We first split the dataset using a random sampling procedure in SPSS to create a training dataset and a test dataset. The training dataset, which included approximately a third of responses (*n* = 122) was used to conduct an EFA and determine the dimensional structure of the scale. Based on recommendations from experts, this sample size is adequate for the purposes of the analysis (45, 46). We conducted the EFA separately for (a) attitudes, beliefs, and emotions items, and (b) knowledge items. This was necessary because the attitudes, beliefs, and emotions were measured using a Likert scale resulting in polytomous ratings, and knowledge items provided dichotomous responses. In addition, the latter items were conceptually different from the rest, as they were designed to measure knowledge about the nature of CRC, its risk factors, early detection, and prognosis. The test dataset consisted of approximately two-thirds of responses (*n* = 283) and was used to conduct a CFA based on a multidimensional Rasch model for validation purposes and to further evaluate the CCLS–U’s psychometric properties. This sample size is also consistent with prior recommendations in the literature [see (47–49)].

### Exploratory factor analysis

Prior to conducting the EFA, we tested relevant statistical assumptions. The Kaiser-Meyer-Olkin measure of sampling adequacy was 0.70, which is acceptable for conducting factor analysis (50, 51). Moreover, when converted to a Chi-square statistic, Bartlett’s test of sphericity yielded a *p* < 0.0001, indicating unequal variances. These statistics provided support for the suitability of the data for the EFA. We subsequently conducted the EFA on 36 items related to attitudes, beliefs, and emotions. Based on an oblique rotation, a scree plot included 24 factors with eigenvalues >1, ranging from 8.28 to 1.03, although there was an elbow after the fourth factor. From the eighth factor forward, the increase in eigenvalues was very small. We also conducted a parallel analysis using the *psych* package in R. Results suggested six factors or four components to be extracted. Because we only had 36 items in total, we investigated the structures with three, four, and five factors.

After reviewing these factor structures, the four-factor solution emerged as the most interpretable. Through an iterative process of deleting items that did not meet retention criteria and rerunning the EFA, eight items with factor loadings below 0.35 were removed, yielding a 28-item solution. Factor I included five items focused on attitudes, beliefs, and emotions about medical science; Factor II was comprised of 10 items that tapped into attitudes, beliefs, emotions, and dispositions toward prevention; Factor III included four items related to attitudes and beliefs about cancer; and Factor IV contained nine items related to emotions about cancer. Given that two factors had low item counts, and consistent with the conceptualization of the study, we combined Factors I and II to represent attitudes, beliefs, emotions, and dispositions related to medical science and prevention (heretofore called Factor I; 15 items), and combined Factors III and IV to reflect attitudes, beliefs, and emotions associated with cancer (heretofore called Factor II; 13 items). We subsequently conducted another EFA on the new set of items and explored a two-factor structure solution. Two items had factor loading below 0.35; these two items were subsequently removed. Also, in the new structure, three items that originally had higher loadings on the second factor showed higher loadings on the first factor. Thus, the final two-factor structure included 17 items measuring Factor I and 9 items on Factor II. To these two factors we added a third factor measuring knowledge through a separate EFA. This last factor, which originally included 33 items, had 13 items removed; the remaining 20 items comprised Factor III. Eight items were removed due to low factor loadings and another five items due to unprecise wording that may result in a true or false correct response (e.g., “A risk factor for CRC is… Having a personal medical history of other types of cancer”—where the answer is dependent on the type of cancer). Each of the three factors is described next.

#### Factor I: disposition toward CRC prevention and diagnosis

Upon finalizing the EFA, Factor I consisted of 17 items. However, in the process of conducting the CFA, we obtained item–total correlations and EAP reliabilities for review. We found two items with very low item–total correlations (*r* = .062 and *r* = .135); these items did not fit the structure well. Subsequently, we removed these two items and saw an increase in all EAP reliabilities, evidence of an increased model data fit. The final Factor I subscale, which comprises 15 items, assesses the respondents’ disposition toward CRC prevention including relevant attitudes, beliefs, emotions, and behavioral intentions. Sample items include “If I noticed a colorectal cancer symptom, I would go to the doctor to get it checked” (an anticipatory behavior toward prevention), “How important is it to get a fecal blood test?” (a belief about screening), and “If I found out I had colorectal cancer, I would feel anxious” (an anticipatory emotion related to diagnosis). We conceptualize these items as measuring the most proximal influences on screening behaviors, and therefore the most likely to affect CRC outcomes.

#### Factor II: attitudes, beliefs, and emotions about cancer and CRC

This subscale includes nine items centered on attitudes, beliefs, and distressing emotions associated with cancer more generally and CRC in particular. Focusing on symptoms, diagnosis, and treatment, the items assess perceptions that might negatively influence individuals’ decisions to obtain CRC screenings. Sample items include “I dislike talking about cancer” (an attitude about cancer), “How painful do you think colonoscopies are?” (a belief about CRC screening), and “I would feel sad if I had to tell a family member that I have cancer” (an emotion related to cancer). We conceptualize this factor as measuring constructs that influence screening, although their effects are not as direct as perceptions related to prevention.

#### Factor III: knowledge

This subscale includes 20 items that measure knowledge about CRC risk factors, symptoms, screening processes, and the nature of the condition. Risk factors assessed include some that are true (e.g., “Eating grilled red meat with charred fat”) and some that are false (e.g., “Having anal sex”). Other knowledge items include “It is possible to have colorectal cancer without symptoms” (measuring knowledge about symptoms; true), “A person needs to have a fecal blood test only when something is unusual in her/his feces” (measuring screening knowledge; false), and “It is possible to develop polyps in the intestine” (measuring the nature of CRC; true). Knowledge items are posited to form the basis for associated beliefs that may influence CRC screening.

Factor loadings for Factors I and II are shown in Table 1; factor loadings for Factor III are shown in Table 2. After establishing the factors, we conducted a CFA using Rasch measurement theory for validation purposes and to further assess the CCLS–U’s psychometric properties.

**Table 1:** EFA results for Factors I and II.

Item content by factor	Factor loadings	
	I	II
Factor I: Disposition toward CRC prevention and diagnosis		
Doctor effectiveness	**0.52**	0.09
Medicine effectiveness	**0.50**	0.01
Worried about caring for family if diagnosed	**−0.39**	0.41
Worried about caring for self if diagnosed	**−0.35**	0.22
Can avoid CRC	**0.62**	−0.20
Anxious about diagnosis	**−0.40**	0.38
Attentive health care provider	**−0.54**	−0.05
Would get symptom checked	**0.52**	0.08
Embarrassed digital rectal exam	**0.42**	0.33
Interested in CRC information	**0.52**	−0.09
FOBT is important	**0.74**	−0.01
Colonoscopy is important	**0.85**	0.00
Cancer is a divine punishment	**0.52**	0.17
Cancer treatment is worse than the disease.	**0.35**	0.28
Sad if diagnosed	**−0.48**	0.46
Factor II: attitudes, beliefs, and emotions about cancer and CRC		
Dislikes talking about cancer	0.38	**0.48**
Pain with colonoscopy	0.01	**0.36**
Worry about removing intestines	−0.07	**0.50**
Burden to loved ones	0.10	**0.51**
Afraid of cancer treatment	−0.01	**0.70**
Uncomfortable looking at stool	0.46	**0.58**
Uncomfortable tracking bowel movements	0.35	**0.62**
Sad telling others about diagnosis	−0.25	**0.70**
Uncomfortable placing stool sample in refrigerator	0.17	**0.45**

*N* = 405. CRC, colorectal cancer. Item wording has been abbreviated. The full scale is available by request from the LB. Bold values represent loadings for the corresponding factor.

**Table 2:** EFA results for Factor III.

Item content	Factor loading
Knowledge about CRC risk factors	
Age over 50	**0.56**
CRC family history	**0.56**
Personal medical history	**0.44**
Thinking about CRC	**−0.43**
Drinking alcohol	**0.44**
Lack of hygiene	**−0.50**
Lack of exercise	**0.68**
Obesity or overweight	**0.83**
Fatty foods	**0.47**
Red meat with charred fat	**0.48**
Anal sex	**−0.71**
Sex with someone diagnosed	**−0.67**
Other CRC knowledge	
Asymptomatic	**0.49**
Symptom: change in bowel habits	**0.41**
Can develop polyps in intestine	**0.53**
Symptom: bloody stool	**0.53**
FOBT only when something unusual	**0.49**
FOBT can find problems	**0.62**
Colonoscopy done to find tumor	**0.77**
Colonoscopy can find a polyp	**0.77**

*N* = 405. CRC, colorectal cancer. Item wording has been abbreviated. The full scale is available by request from the LB. Bold values represent loadings for the corresponding factor.

### Confirmatory factor analysis

The use of Rasch measurement theory in scale development has grown significantly in recent years. It is currently widely applied in the United States and internationally across various fields, including the medical and social sciences [e.g., (52–54)]. Rasch measurement theory, in contrast to classical test theory, releases the assumptions that non-Rasch techniques hold [e.g., assuming equal item difficulty across all items, equal jumps across various points in a rating scale, and equal ability to answer all items by the test taker (55)]. Because we expected correlated latent constructs, we used a multidimensional Rasch model to increase estimation precision. In addition, we examined a multidimensional partial credit Rasch model to make estimations across items scored using different scale structures. The model-data fit for each individual item was assessed using item weighted fit (MSE).

To evaluate fit indices, we followed the criterion proposed by Engelhard & Wind (56), 0.50 < fit <1.50. Results revealed that none of the items violated this standard (range: 0.75–1.33). Also, the ideal value, 1.0, fell inside the 95% confidence intervals of the weighted fit statistic for all items. Thus, results showed a good fit of items to the model.

Reliability was computed using maximum likelihood estimation with expected *a posteriori* [EAP (57, 58)]. This reliability quantifies the amount of uncertainty in the measurement process and is interpreted similarly to traditional reliability indices (e.g., Cronbach’s alpha). In contrast to traditional indices, EAP reliability is based on the variance of latent measures. For the 44-item multidimensional scale, we obtained EAP values as follows: Factor I = .71; Factor II = .70; Factor III = .70. Overall, reliabilities were .70 or above, presenting an acceptable fit and indicating good quality of the measuring instrument.

### Psychometric properties

#### Content and face validity

To develop the items, we first engaged in a deductive process, defining the universe of items broadly. We purposefully surveyed various areas associated with health literacy with respect to CRC including (a) beliefs, attitudes, and emotions related to cancer more generally; (b) beliefs, attitudes, and emotions related to CRC signs and symptoms, screening exams, treatment, and prognosis; (c) experiences and expectations related to physician–patient interactions, and (d) knowledge of CRC signs and symptoms, screening exams, treatment, and prognosis. For all areas, we developed items in a systematic manner, consistent with recommendations from Cronbach and Meehl (59). We then asked public health experts to review the items, obtaining support for face validity. Subsequently, we conducted focus groups with community members and health care professionals to further validate these domains and ensure broad coverage of the topic, consistent with expert recommendations to enhance content and cognitive validity [e.g., (44)].

#### Criterion-related validity

As part of the psychometric analyses, we report on criterion-related validity, which is comprised of predictive, concurrent, convergent, and discriminant validity. *Predictive validity* indicates the strength of the relationship between the current scores and criterion scores obtained at some point in the future; these scores are typically assessed using a gold standard measure of a theoretically related construct (60). *Concurrent validity* refers to the degree of association between results using the newly constructed measure and the results of an established measure administered within a similar time frame. In turn, *convergent validity* provides information about the relationship between test scores and other measures of the same construct or a related construct (60). We were unable to examine these types of validity in this study because there are no measures available to assess CRC-related health literacy, or health literacy more broadly, in Uruguay. Given the lack of a “gold standard,” we had no way of comparing scores on the current measure to those of more established measures of the construct.

To assess *discriminant validity*, which refers to a lack of association across different constructs, we examined interscale correlations. Given our theoretical conceptualization, we expected a strong positive correlation between Factor I (i.e., Disposition Toward CRC Prevention and Diagnosis) and Factor II (i.e., Attitudes, Beliefs, and Emotions about Cancer). A high correlation would reflect that individuals’ greater disposition toward cancer prevention would be associated with holding favorable attitudes, beliefs, and emotions about cancer and CRC. In addition, we anticipated there would be a strong positive correlation between Factor I (i.e., Disposition Toward CRC Prevention and Diagnosis), and Factor III (i.e., Knowledge). A high correlation would suggest that individuals’ favorable disposition toward cancer prevention would be related to having knowledge about risk factors, symptoms, screening processes, and the nature of the condition. As expected, interscale correlations were higher between Factors I and II, *r* = 0.72, *p* < 0.001, and Factors I and III, *r* = 0.57, *p* < 0.001, than between Factors II and III, *r* = 0.29, *p* < 0.001. The correlations were consistent with the expected strength and direction, providing support of discriminant validity in this sample.

## Discussion

Initial evaluation of the CCLS-U supports its psychometric validity and reliability when used with a diverse Uruguayan national sample. To our knowledge, this mixed-methods study represents the first attempt to develop an empirically based measure to assess cultural and conceptual components of CRC health literacy in a Latin American country. Importantly, a psychometrically sound instrument grounded in the health literacy model allows for further advances in theory, research, and practice aimed at reducing CRC morbidity and mortality. In the next paragraphs we discuss strengths of the study, place results within the larger context of the literature, note limitations, and provide specific implications for future work in this area.

A particular strength of the study was its heterogeneous sample with good representation of individuals who would be expected to score high and score low, given their diverse demographic characteristics (23, 61, 62). In addition, the variation across geographic regions (e.g., urban areas, rural border towns) ensured representation of a range of cultural contexts within a relatively small country. We intentionally included an equivalent number of participants who identify as women and as men, given that CRC affects everyone, regardless of sex or gender identity.

The rigorous and systematic process followed to develop the measure is another strength of the study. Based on steps designed to enhance psychometric validity [e.g., (62)], we first conducted a thorough review of the literature, generated a comprehensive list of items, and refined them via focus groups and consultation with experts in the field. As intended, by following these recommended processes, the final measure showed good indices related to EAP reliability, content and face validity, and discriminant validity.

Specifically, Factor I captures dispositions toward cancer prevention and diagnosis including beliefs, attitudes, and emotions related to cancer screening and diagnosis. Factor II represents beliefs, attitudes, and emotions related to cancer in general and CRC in particular. In turn, Factor III includes knowledge items related to cancer risk factors, symptoms and signs, and prognosis. These factors are consistent with some found in prior scale development studies in cancer prevention. A psychometric validation of a measure assessing cultural constructs related to breast and cervical cancer screening in Latina populations yielded one factor related to disposition toward cancer prevention (i.e., negative beliefs about health professionals, sociocultural deterrents to screening), and a second factor related to attitudes and beliefs about cancer [i.e., catastrophic disease expectations (63)]. In a psychometric evaluation of a scale measuring aversion to CRC screening, one subscale encompassed related emotions (64). Our findings are also consistent with extant conceptualizations of disease-specific health literacy [e.g., (65)] and cultural and conceptual knowledge [e.g., (26, 63)].

### Limitations of the study

Due to the lack of related measures designed for the Uruguayan population, we could not assess predictive, concurrent, and convergent validity. As measures become available, it would be important to conduct a further assessment of criterion-related validity. An additional limitation is that as a volunteer sample, participants who had the time and interest to complete the measures could be overrepresented. However, this was mitigated by the fact that about two-thirds of participants were still active in the workforce. Also, participants represented a wide range of demographic factors as well as variation in screening status. Beyond these limitations, this is the first study to examine CRC health literacy in Uruguayan populations, with implications for theory, research, and practice. We discuss these next.

### Implications for theory and research

The CCLS-U is the first instrument designed to assess an integral component of health literacy that heretofore could not be measured—cultural and conceptual knowledge related to CRC. Thus, when administered along with measures of print literacy, oral literacy, and numeracy, a comprehensive assessment of health literacy will be achieved. Theoretical conceptualizations subsequently may be advanced to understand the relation between health literacy, screening adherence, and health promotion more generally. For example, if systematic differences are uncovered in CRC screening uptake, psychosocial factors associated with this inequity may be examined using the measure. In addition, the measure can facilitate the examination of extant conceptual models that contemplate the influence of cultural and conceptual knowledge on CRC behavioral outcomes. Using informed decision-making theory as a framework, researchers may assess personal and cultural factors that influence decision-making with respect to cancer screening. For instance, researchers could assess participants’ culturally based perceptions of CRC screening as well as their knowledge of CRC screening risks and benefits. This would facilitate an evaluation of the role of culturally based factors in patients’ process of weighing the risks and benefits of screening. Thus, with these data, researchers may identify additional information and/or supports needed by the patient to facilitate decision-making with respect to screening (66).

The measure may be used, as well, to identify psychosocial factors that discriminate among individuals who are up-to-date and overdue for screening such as knowledge, beliefs, attitudes, and emotions that need to be addressed through intervention. Because sex and gender interactions influence decisions related to health and well-being (67), examining differences across these factors would be important. The measure may also be used to subsequently evaluate intervention outcomes and identify the most promising approaches to increasing screening rates, assisting in the process of optimizing health promotion resources. To understand factors that contribute to health literacy changes across time, the CCLS-U may be used in longitudinal studies. Information gathered through these studies can help further refine theoretical frameworks and interventions. In addition, the long-term efficacy of interventions and/or national campaigns may be evaluated over time using the measure.

Future directions for research may also include adapting the CCLS-U for use with new populations. This process would require an extensive review of the literature on knowledge, beliefs, attitudes, and emotions related to CRC for the population of focus. Adaptations may include revising the phrasing of questions as well as adding or eliminating certain items given the cultural and psychosocial characteristics of the sample. We recommend conducting focus groups to assess the cultural relevance and clarity of revised items prior to finalizing it. As part of the focus groups, participants may be asked to say out loud what they understand by certain items. This cognitive debriefing exercise would allow researchers to evaluate whether their intent in designing the item corresponds to the way participants understand it.

In cases where the new measure is in a different language, focus group participants should be asked about the appropriateness of translated concepts, especially words and ideas that are challenging to convey in the target language. In all cases, researchers would be well-advised to be mindful of local dialects or cultural nuances by geographic locale. In the event that revisions are extensive, we recommend conducting psychometric testing to examine the reliability and validity of the revised measure with the new population.

Similarly, another future research direction would include adapting the CCLS-U for use with other cancers for which early detection is also key to survival. This process would require an extensive review of the literature on health literacy, knowledge, beliefs, attitudes, and emotions related to the specific cancer examined and its screening methods. Again, adaptations may include revising the content of questions as well as adding or eliminating certain items given the nature of the cancer, the population of interest, and the type of screening procedure (e.g., a focus on women and Pap smear testing to examine cervical cancer health literacy). Similarly, if extensive revisions are made, it would be important to examine the psychometric properties of the revised measure.

In future studies, researchers may also explore shortening the current measure to create a checklist that a patient could easily complete while waiting to be seen by a health care professional. Key items may act as a screener and offer clinicians information they can use to tailor psychoeducational messages (e.g., focus on clearing up incorrect information) during the medical appointment.

### Implications for practice

The CCLS-U can help identify populations at risk due to low screening rates. This information can be used to advocate for the allocation of scant resources to medically underserved populations (e.g., individuals with low health literacy), thereby optimizing impact. Specifically, the measure may be used to examine possible associations among sociodemographic factors (e.g., region of the country, family history of CRC, formal education, sex, gender), cultural and conceptual knowledge, and screening behaviors. For example, an analysis of score differences across *departamentos* may yield information about underserved areas where screening rates are low and public health programs are needed. Similarly, the measure may be used to understand the unique educational needs of traditionally underrepresented groups, such as Uruguayans of African descent and individuals with disabilities. In this way, the measure can serve used as a tool to uncover, understand, and address linkages between social determinants of health and cancer screening outcomes.

In addition, the CCLS-U may help identify facilitative factors and barriers to CRC screening experienced by a specific group (e.g., women, men). With these data, practitioners may tailor health promotion messages and referrals that, importantly, may lead to higher CRC screening rates. This is consistent with recommendations to promote cultural sensitivity through attention to deep structure by crafting messages that address the intended audience’s psychosocial, cultural, and environmental perceptions related to health and illness (68, 69). Tailored messages would be disseminated through psychoeducational interventions such as educational materials, workshops, and screening reminders. These interventions may be directed at communities, individuals at risk or overdue for screening, and/or their families. In addition, health care professionals may benefit from training opportunities to learn about knowledge, beliefs, attitudes, and emotions that would be timely to address with a specific population.

Specifically, we found that Factor I relates to individuals’ attitudes, beliefs, emotions, and dispositions toward prevention and diagnosis; these constructs are amenable to change through psychoeducational programming, which makes it critical to measure them for future intervention. Items comprising Factor II relate to attitudes, beliefs, and emotions about cancer, which may be used to tailor culturally relevant interventions. Factor III consists of items measuring CRC knowledge such as knowledge about the nature of CRC, its risk factors, early detection, and prognosis. Having the ability to measure knowledge enables researchers and practitioners to examine increases in knowledge following targeted intervention.

We also recommend questioning the status quo, which is the common practice of giving psychoeducational workshops on early screening without incorporating tailored approaches to modify each of the constructs—knowledge, beliefs, attitudes, and emotions. For example, patients who are afraid of cancer treatment (an emotion) might benefit from an intervention that requires them to examine the underlying cultural messages giving rise to this emotion. In turn, patients who lack information might benefit from exposure to information that enhances their knowledge about CRC, such as dietary risk factors and needed dietary changes.

## Conclusion

In this study, we described the development and psychometric validation of the CCLS-U, a newly developed scale to assess culturally based factors that influence CRC screening behaviors. Initial data suggest the viability of the scale, which can be used to advance theory, research, and practice in this area. As additional measures of health literacy and related factors become available for the Uruguayan population, the scale may be further developed by examining its convergent validity and assessing discriminant validity in alternative ways. Moreover, as the scale is implemented, its predictive validity may be examined. We present detailed strategies to adapt the CCLS-U for use with other populations. In addition, we make recommendations for revisions to develop a measure focused on other cancers in which early detection is also key to survival. We are hopeful that this scale will become an important tool in promoting health, diminishing the cancer burden, and avoiding premature death in populations at high risk for CRC.

## Data availability statement

The dataset generated and analyzed during the current study is not publicly available due to the fact that participants did not give written consent for their data to be shared publicly.

## Ethics statement

This study was approved by the University of Miami Human Subjects Research Office and the Instituto Nacional del Cáncer in Uruguay. The study was conducted in accordance with local legislation and institutional requirements. The study involved minimal risk. The institutional review board/ethics committee waived the requirement of written informed consent for participation from the participants; verbal consent was obtained instead.

## Author contributions

LB and MR made substantial contributions to the conception, design, interpretation of data, and manuscript writing. JW made substantial contributions to the analysis and interpretation of data. SS, DL, BS, and MB made substantial contributions to the conception, design, and acquisition of data. All authors contributed to the article and approved the submitted version.

## Funding

Funding for travel to Uruguay was given to LB by the University of Miami Institute for the Advanced Study of the Americas.

## Conflict of interest

The authors declare that the research was conducted in the absence of any commercial or financial relationships that could be construed as a potential conflict of interest.

## Publisher’s note

All claims expressed in this article are solely those of the authors and do not necessarily represent those of their affiliated organizations, or those of the publisher, the editors and the reviewers. Any product that may be evaluated in this article, or claim that may be made by its manufacturer, is not guaranteed or endorsed by the publisher.
